# Rapid Shifts in Relative Abundance Obscure Temporal Diversity Changes in a Metacommunity

**DOI:** 10.1002/ece3.71694

**Published:** 2025-07-02

**Authors:** William Godsoe, Warwick J. Allen, Lauren P. Waller, Barbara I. P. Barratt, Sarah P. Flanagan, Zachary H. Marion, Jason M. Tylianakis, Elena Moltchanova, Ian A. Dickie

**Affiliations:** ^1^ Lincoln University Lincoln New Zealand; ^2^ School of Biological Sciences University of Canterbury Christchurch New Zealand; ^3^ Bioprotection Aotearoa Lincoln University Lincoln New Zealand; ^4^ AgResearch Invermay Research Centre Mosgiel New Zealand; ^5^ Department of Botany University of Otago Dunedin New Zealand; ^6^ Bioprotection Aotearoa, School of Biological Sciences University of Canterbury Christchurch New Zealand; ^7^ School of Mathematics and Statistics University of Canterbury Christchurch New Zealand

**Keywords:** beta diversity, community assembly, Hill numbers, mesocosm, metacommunity, scale

## Abstract

Changes in biodiversity reflect processes acting at multiple spatial scales, from local to global, among habitats and within communities. This complexity makes it difficult to measure mechanisms that have traditionally interested ecologists, such as environmental filters. To resolve this, we propose an approach to partition temporal changes in biodiversity into contributions from selection at multiple scales. We applied this approach to study changes in the biodiversity of invertebrate herbivores from a large‐scale, plant community experiment. Though the experiment was designed to foster distinct insect communities due to differences in host plants, our approach showed that selection among these treatments was a negligible facet of diversity change. These effects were swamped by rapid changes in relative abundances of aphids due to both immigration and selection across the metacommunity. More broadly, our work highlights how total change in biodiversity across a biogeographic region can be partitioned into logically distinct mechanisms.

## Graphical abstract

Using an analogy to natural selection, we propose a novel approach to tease apart processes shaping biodiversity at multiple spatial scales. Using data from a metacommunity study of insect herbivores, we show selection among species was more important than selection between treatments or between communities. This effect is easy to miss when studying overall changes in diversity over time.

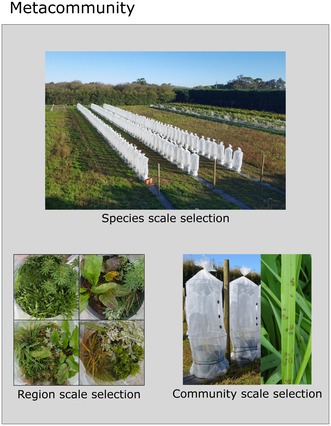

## 1 Introduction

Biodiversity describes the variety of living things on Earth, including the richness (number of species present) and evenness (similarity of the relative abundances) of different species. There is great interest in understanding how biodiversity changes over time; however, this goal is complicated by the fact that biodiversity is spatially structured (Whittaker 1956; Chase et al. 2018). For instance, starting from broad spatial scales, we may define gamma diversity, (*γ*) as the overall biodiversity of a region. Measures of gamma diversity can be separated into components of alpha diversity (*α*), the average biodiversity within communities (see Appendix S2: Table S1), and beta diversity (*β*), which describes dissimilarity among communities (Jost 2007). Even this familiar terminology is a simplification. In practice, changes in diversity can reflect processes acting at several additional scales necessitating further subdivisions. For example, McGill et al. (2015) showed that observations of diversity change over time can lead to different conclusions at four distinct spatial scales (1) globally, (2) within biogeographic regions, (3) within metacommunities, and (4) within communities.

Given this complexity, it is challenging to understand which mechanisms lead to broad‐scale changes in biodiversity (Connor and Simberloff 1979; Kraft et al. 2015; Cadotte and Tucker 2017; Godsoe et al. 2023). Many broad‐scale patterns in ecology are thought to depend on interactions among species (MacArthur 1965; Gotelli et al. 2010; Wisz et al. 2013; Louthan et al. 2015; Godsoe et al. 2017), but it is not clear how to detect the effect of these interactions on temporal changes in diversity (Legendre 2019; Magurran et al. 2019). Some sense of the challenge can be gleaned from the many studies that seek to separate the consequences of different environmental “filters” that restrict biodiversity in particular communities, such as competition among species versus the abiotic environment. Though many studies have sought to measure interactions between these two mechanisms, a literature review by Kraft et al. (2015) showed that only 15% of studies considered evidence that species could survive the abiotic environment in the absence of biotic interactions. It was much more common to simply assume that the effects of the abiotic environment and biotic interactions could be separated.

Many existing studies of environmental filters focus on plant diversity, but further complexities are likely to emerge in groups such as herbivorous insects. Across biogeographic regions, insect diversity is structured by broad‐scale environmental gradients such as elevation (Whittaker 1952). Nested within these broad‐scale gradients, the fitness of herbivorous insects is mediated by interactions with other insects and host plants (Van Zandt and Agrawal 2004). There is therefore a need to understand the interplay of these processes across scales (Thompson and Cunningham 2002). In insects, population growth can be rapid and competition is difficult to analyze because it is often asymmetric (Kaplan and Denno 2007). In view of the current difficulties in teasing apart the role of different environmental filters in shaping temporal patterns of diversity, we suggest an alternative: What if we try to quantify the effect of selection at different levels instead of competition or filtering?

When studying mechanisms shaping biodiversity, it is natural to focus on relative abundances, rather than absolute abundances (Godsoe et al. 2023); the reason being that measures of species diversity depend on relative abundances (Patil and Taillie 1982; Jost 2007). Using the terminology suggested by Vellend (2010), the logical way to study changes in relative abundances is to analyze selection among species. Selection occurs when some species have a higher relative fitness than others (Vellend 2010; Mallet 2012; Vellend 2016, McPeek 2017a; Viana and Chase 2019). A major benefit of this approach is that diversity can be sensitive to selection, even when it is insensitive to the effects of competition (Godsoe et al. 2023). In Vellend's terminology, selection is a “high‐level process” which can aggregate the effects of many “low‐level processes” including competition, tolerance of environmental stressors, avoidance of predators, or any number of other factors (Vellend 2016: section 4.3). Many other ecological mechanisms indirectly produce selection, including other types of interactions among species and density‐independent growth (Mallet 2012; McPeek 2017a). A number of tools are available to quantify the effects of selection in nature (Kingsolver et al. 2001; Hairston et al. 2005; Ellner et al. 2011).

When studying a biogeographic region, it is useful to start by considering selection among species. For example, Bode et al. (2011) studied coexistence among fish species on the Great Barrier Reef. They showed that differences in dispersal distance among species promoted coexistence among competitors. The reason for this is that long‐distance dispersers can specialize on distant habitat patches, while short‐distance dispersers can specialize on nearby habitat patches. This mechanism acts among species across the entire biogeographic region (e.g., the Great Barrier Reef) and should increase gamma diversity.

In addition to species‐level selection, diversity can also change when individuals in some habitats are more successful than individuals in other locations. For example, some plant species in the Himalayas produce many offspring in low elevation habitats (Klimeš and Doležal 2010), such that success in one region and failure in another increases beta diversity. Beta diversity may also increase due to selection among communities. For example, a species may produce many offspring in one community but die off in another. Though selection may occur at different levels (i.e., species, habitats, communities), at present we lack the tools to disentangle its effects on diversity.

Here we seek to define and quantify the effect of selection at different levels in a metacommunity: selection among species, selection among individuals in different habitats, and selection among individuals in different communities. The goal of our approach was to measure how fitness differences change components of diversity in a metacommunity with multiple levels. This will lead to a series of terms describing how diversity responds to selection based on species, habitat, and community of origin. To measure these terms, we first explicitly label individual contributions to relative abundance. We then quantify individual contributions to alpha, beta, and gamma diversity. We then derive an expression for change in diversity. Finally, we modify the expression for diversity change to consider how differences in relative fitness at different levels alter diversity.

**FIGURE 1:**
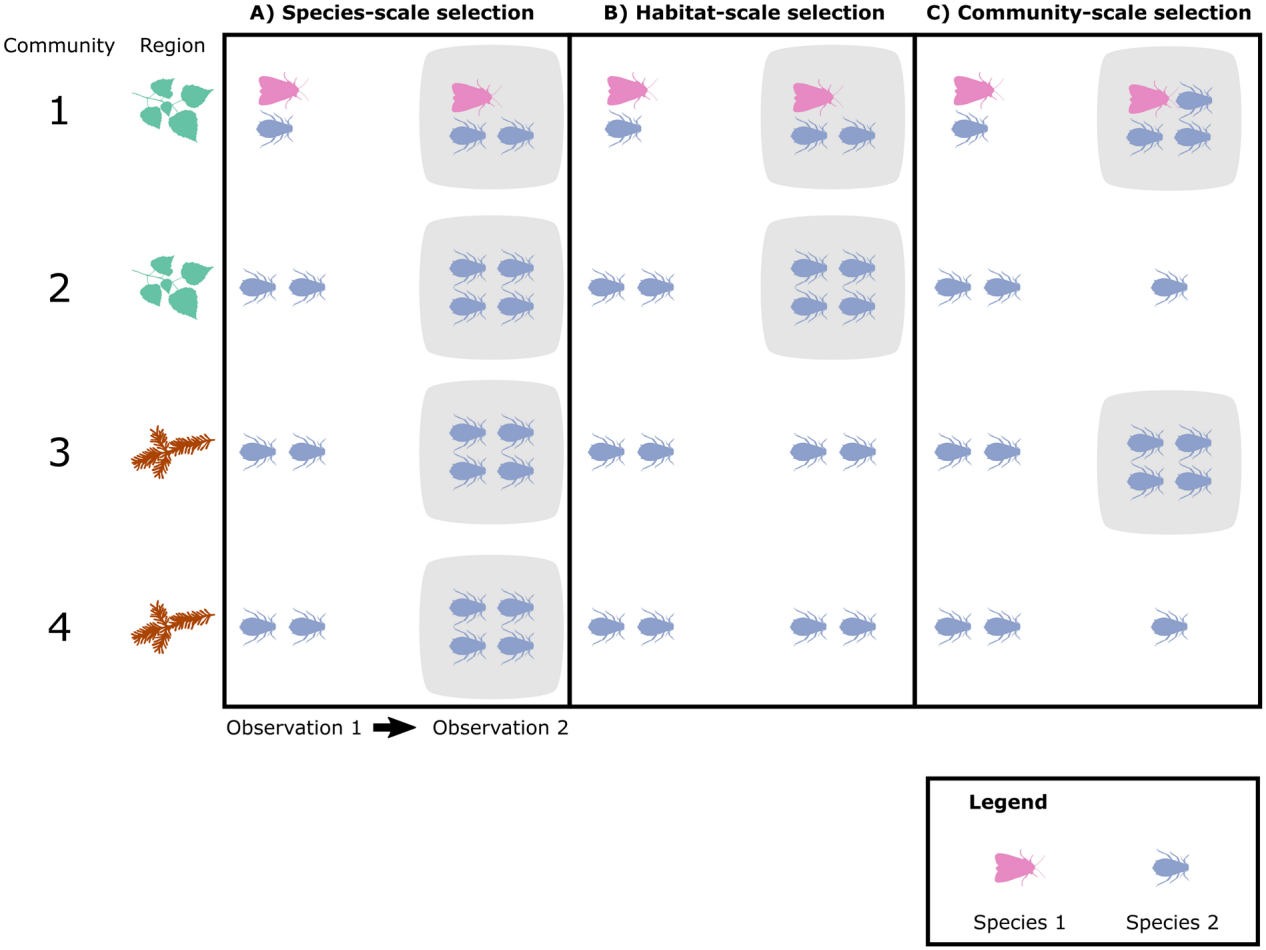
Changes in biodiversity can result from selection at different scales. Each panel shows a biogeographic region consisting of four communities divided into two habitats, with Communities 1 and 2 dominated by deciduous plants and Communities 3 and 4 dominated by coniferous plants. Species 1, a moth (purple) is restricted to Community 1 and Species 2, is a broadly distributed aphid (blue). Each panel shows changes between two observations. (A) Illustrates species‐scale selection, where Species 2 increases in relative abundance across all communities (highlighted in gray). (B) Illustrates habitat‐scale selection, where the blue species increases in the deciduous dominated habitat 1 relative to the coniferous dominated Habitat 2. (C) Illustrates community‐scale selection, where Species 2 increases in some communities relative to others. At present, it is not clear how to disentangle the effects of selection at each scale.

To illustrate our approach, we partitioned change in biodiversity using data from a large‐scale, plant–herbivore community experiment (Allen et al. 2021). The experiment can be divided into distinct “habitats,” which are represented by treatments containing different combinations of host plants. In turn, these habitats are divided into local communities: individual mesocosms with experimentally inhibited (though not completely eliminated) herbivore dispersal among them. This design was established to consider changes in insect communities at different scales (Figure 1). Given the experimental design, we expected diversity to change dramatically due to strong selection among habitats. Instead, we found that selection among habitats was a negligible source of diversity change. The major drivers of diversity change were outbreaks of several aphid species, which grew in numbers, then rapidly declined. These observations suggest advantages in formally quantifying selection at different scales, as the mechanisms shaping diversity change in metacommunities are easy to conflate.

## 2 The Model

### 2.1 Temporal Changes in Diversity

We consider a biogeographic region where individuals fit into groups at three scales, organized hierarchically. Starting from an observation of diversity in the present, each individual in the metacommunity is a member of one species. Within a species, each individual is a member of one habitat type, and within one combination of species and habitat, each individual is found within one community. We use *n*_*ijk*_ to denote the absolute abundance of species *i* = 1,…,*S*, in habitat *j* = 1,…,*J*, and community *k* = 1,…,*K* (Appendix S1: Table S1 for list of terms). The total number of individuals is *n*_•••_ = ∑_*i*_ ∑_*j*_ ∑_*k*_ *n*_*ijk*_, where • indicates summing across a scale.

The most general measures of diversity, such as Hill numbers, are defined in terms of the probability that an individual belongs to a given type (Jost 2007). Therefore, we define the probability that, out of the biogeographic region, an individual is in species *i*, habitat *j* and community *k* as *p*_*i*jk_ = *n*_*ijk*_/*n*_•••_. The probability that an individual in the metacommunity belongs to species *i* is defined as *p*_*i*••_ = *n*_*i*••_/*n*_*•••*_. This is the relative abundance of species *i* across the metacommunity. Within community *k*, the probability that an individual belongs to species *i* is given by *p*_*i|jk*_ *= n*_*ijk*_ */n*_*•jk*_. This is the relative abundance of species *i* in community *k*. The probability that an individual in species *i* is found in habitat *j* is given by *p*_*j|i*•_ = *n*_*ij*•_/*n*_*i*••_ and the probability that an individual is in community *k* given that we are in species *i* and habitat *j* is given by *p*_*k|ij*_ *= n*_*ijk*_ */n*_*ij*•_.

Diversity changes when there are fitness differences among species, habitats or communities. Typically measures of fitness count all individuals equally (Frank 2012a). Therefore, we weight all individuals equally in our calculations of diversity. Measures based on Shannon entropy are recommended when individuals are weighted equally in a metacommunity (Jost 2007). Other diversity indices such as Gini‐Simpson's, are not recommended because they can produce the paradoxical conclusion that alpha diversity is higher than gamma diversity (Jost 2007).

For Shannon entropy, each diversity component can be decomposed into contributions of each individual in a metacommunity (Jost 2007). We will denote these individual contributions to diversity as $z_{\mathrm{ijk}o}$, with the _o_ indicating one of alpha, beta, or gamma. Godsoe et al. (2022) showed that, for gamma diversity, individual contributions measure a species' rarity across the metacommunity, using *z*_*ijkγ*_ = −log (*p*_*i*••_). For alpha diversity, individual contributions measure a species' rarity in its local community *z*_*ijkα*_ = −log (*p*_*i*|kj_). For beta diversity, individual contributions represent the difference between contributions to gamma and alpha diversity (*z*_*ijkβ*_= *z*_*ijkγ*_ ‐ *z*_*ijkα*_).

Each diversity component (i.e., alpha, beta or gamma) is then the average of the contributions from every individual in the community (Jost 2006, 2007):

(1) $$H_{o}^{\prime }=\sum_{i}\sum_{j}\sum_{k}p_{\mathrm{ijk}}z_{\mathrm{ijko}}.$$

The change in each diversity component over time is the difference between the future ($H_{o}^{\prime }$) and present observation of diversity:

(2) $$∆H_{o}=H_{o}^{\prime }‐H_{o}.$$

So far, we have defined temporal change in diversity, but we have said very little about the mechanisms that shape this change. To tease these terms apart, we will develop a revised partitioning:

(3) $$∆H_{o}=\text{Selection}\;{\text{terms}}_{o}+I{\text{mmigration}}_{o}+{\text{transmission}}_{o}$$

Equation (3) states that total change in diversity represents selection at different levels in a metacommunity, immigration into the metacommunity and a final term to account for nonlinear shifts in diversity as species' relative abundances change.

The principal innovation of the current manuscript is to tease apart the effects of selection at multiple levels. This builds on a previous study by Godsoe et al. (2022), which quantified the effect of selection among species, but did not consider selection at other levels. To develop this partitioning, we first separate contributions to changes in diversity from immigrants, then consider the consequences of fitness on diversity at different levels throughout the metacommunity.

### 2.2 Separating the Effects of Immigration on Diversity Change

To understand how fitness changes diversity, we first must separate individuals that have recently immigrated into a given community. Immigrants are new to a particular community and do not contribute to fitness differences. We denote the relative abundance of immigrants with *q'*, for example $q_{\mathrm{ijk}}^{\prime }$, represents the probability that a recently immigrated individual in the metacommunity belongs to species *i* habitat *j* and community *k*. We define the proportion of individuals that are immigrants across the metacommunity as φ. We label all other individuals as residents of the previous community. We denote the relative abundance of residents with *p'* for example $p_{\mathrm{ijk}}^{\prime }$. The proportion of the community comprising such individuals is $\left(1-\varphi \right)$. Appendix S1 shows that Equation (2) can be rewritten as:

(4) $$∆H_{o}=\varphi \underset{\text{Immigrants}}{\underbrace{\left(H_{o,I}^{\prime }-H_{o}\right)}}+\left(1-\varphi \right)\underset{\text{Residents}}{\underbrace{\left(H_{o,d}^{\prime }-H_{o}\right)}}.$$

**FIGURE 2:**
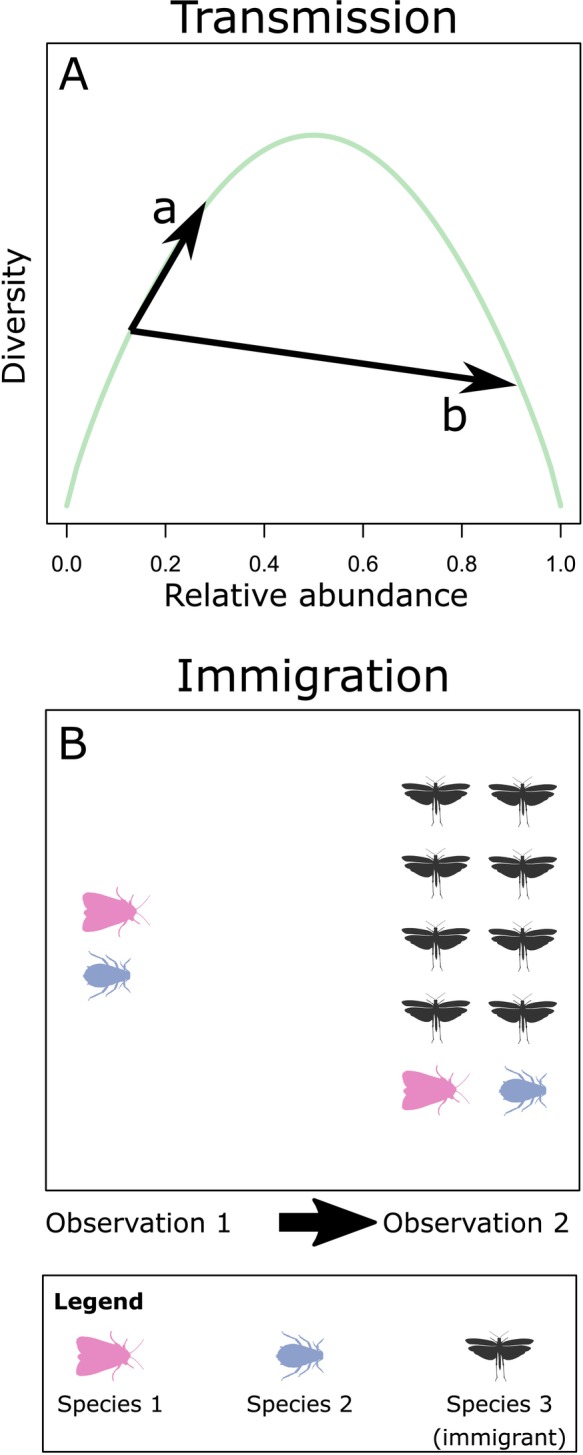
Effects of selection on biodiversity can be obscured by other, less intuitive mechanisms. (A) Shows a plot of biodiversity versus relative abundance for a two species community as a function of the relative abundance of one species (green line). Changes in relative abundance in the same direction sometimes leads to radically different effects. For example, weak selection in favor of a rare species (a) may increase biodiversity, but stronger selection in favor of the same rare species can decrease biodiversity (b). These unintuitive consequences are captured by the transmission term in (Equation 3). (B) Immigration can also change biodiversity. In this panel, the arrival of a new species (black) decreases the evenness of the community, leading to a decline in Shannon entropy from *H* = 0.69 in observation 1 to *H* = 0.63 in observation 2.

Equation (4) assumes that immigrants are distinct from residents (Figure 2). This is important because fitness differences affect the number of residents, but not immigrants. No assumptions are made about the interactions between immigrants and residents. Equation (4) only track individuals that are currently in the metacommunity, therefore emigrants make no contribution to either partition. There is, however, a great deal of latitude in how to identify new immigrants. They may belong to new species or simply be new individuals to the metacommunity. Further generalizations are possible, such as individuals with one parent that is an immigrant, and one parent that is a resident. However, the book keeping in these cases is substantially more complex, and we refer interested readers to (Kerr and Godfrey‐Smith 2009).

### 2.3 Separating the Effects of Selection at Different Scales

We are now ready to consider how fitness differences across the metacommunity alter diversity. In Equation (2) we defined diversity using a single number to describe the probability of different types of individuals ($p_{\mathrm{ijk}}$). This single number reflects information at different scales in the metacommunity (species, habitat, and community). To tease apart changes in diversity across these scales, we use the definition of conditional probability to decompose this term into contributions at each scale (Ross 1997):

(5) $$p_{\mathrm{ijk}}=p_{i••}p_{j|i•}p_{k|\mathrm{ij}}.$$

This states that $p_{\mathrm{ijk}}$ is the product of the probability that an individual is a member of species *i*, the probability that it is in habitat *j* given that it is in species *i* and the probability that it is in community *k*, given that it is in species *i* and habitat *j*.

With this definition in hand, we are able to explicitly track changes at each scale in the metacommunity. Appendix S1 substitutes Equation (5) into our definitions of diversity. We use this revised definition to produce a revised term for the difference between residents in the future observation period and diversity in the present (Equation 4):

(6) $$H_{o,d}^{\prime }-H_{o}=\sum_{i}p_{i\text{••}}^{\prime }\sum_{j}p_{j|i\text{•}}^{\prime }\sum_{k}p_{k|\mathrm{ij}}^{\prime }z_{\mathrm{ijko}}^{\prime }\;-\sum_{i}p_{i\text{••}}\sum_{j}p_{j|i\text{•}}\sum_{k}p_{k|\mathrm{ij}}z_{\mathrm{ijko}}$$

This notation emphasizes that diversity change over time reflects contributions of across different scales in the metacommunity. At each scale, probabilities change in response to fitness differences, that is, differences in per‐capita population growth (Frank 2012b; McPeek 2017b). To tease apart these effects we use the discrete time equivalent of the product rule from calculus (Frank 2018). This results in an expression which divides total change in diversity terms representing change in each of the four variables in (Equation 6) ($p_{i••}$, $p_{j|i•},p_{k|\mathrm{ij}},$and $z_{\mathrm{ijk}o}$):

(7) $$\begin{array}{cc} H_{o,d}^{\prime }-H_{o}= & \sum_{i}∆p_{i\text{••}}\sum_{j}p_{j|i\text{•}}\sum_{k}p_{k|\mathrm{ij}}z_{\mathrm{ijko}}+\sum_{i}p_{i\text{••}}\sum_{j}∆p_{j|i\text{•}}\sum_{k}p_{k|\mathrm{ij}}z_{\mathrm{ijko}}\\ & +\sum_{i}p_{i\text{••}}\sum_{j}p_{j|i\text{•}}\sum_{k}∆p_{k|\mathrm{ij}}z_{\mathrm{ijko}}\;+\sum_{i}p_{i\text{••}}\sum_{j}p_{j|i\text{•}}\sum_{k}p_{k|\mathrm{ij}}∆z_{\mathrm{ijko}} \end{array}$$

In deterministic models, the first three terms on the right‐hand side reflect selection at different scales. At the broadest scale, species selection describes the consequences of changes in relative abundance among species (i.e., $∆p_{i••}$) across the entire biogeographic region (Figure 1A). This will increase diversity when species that make high contributions to the diversity component of interest (i.e., have high $z_{\mathrm{ijk}o}$ scores) increase in relative abundance. At the next scale, habitat‐scale selection describes changes within individual species arising due to individuals within one habitat increasing in abundance relative to individuals in other habitats (Figure 1B). This will increase diversity when individuals in habitats with high $z_{\mathrm{ijk}o}$scores increase in relative abundance. At the finest scale, community selection describes the abundance of individuals of one species within one community increasing in abundance relative to other communities in the same habitat ($∆p_{k|\mathrm{ij}}$; Figure 1C). This will increase diversity when individuals in communities with high $z_{\mathrm{ijk}o}$ scores increase in relative abundance. The three scales we have used are sufficient to illustrate our approach, but additional scales can be added using similar methods (Ross 1997; Frank 2012a). Some analyses focus on diversity per unit area (McGill 2011) but this is beyond the scope of the current manuscript. When stochastic effects are present, the selection terms also capture the effects of changes in relative abundance due to chance events (i.e., drift, Rice 2004). Godsoe et al. (2022) outlines a procedure to test the effect of stochastic drift using Monte Carlo simulation.

In addition to selection terms, a fourth term “transmission” is needed. The need for this term is illustrated in Figure 2A, which shows how a small amount of selection (a) increases diversity, but further increasing the strength of selection (b) paradoxically decreases diversity. The reason for this discrepancy is that diversity does not shift linearly with selection, hence the need for the transmission term to account for this gap. Transmission is particularly important when changes in relative abundance are rapid, which can lead to surprisingly small observed changes in diversity (Godsoe et al. 2021; Edmonds et al. 2023).

One potential source of confusion in the partitioning we present is that the units of Shannon entropy are unfamiliar to many ecologists (Cover and Thomas 2012). Fortunately, our partitions of diversity change in Equations (4) and (7) can be exponentiated to convert these expressions into an analysis of Hill numbers. Hill numbers measure the equivalent amount of species richness used to produce a given observation of Shannon entropy (Hill 1973; Jost 2007; See Appendix S1 for details). This also produces measures of beta diversity that are not constrained by the value of alpha diversity (Chao et al. 2012).

## 3 Example Dataset

**FIGURE 3:**
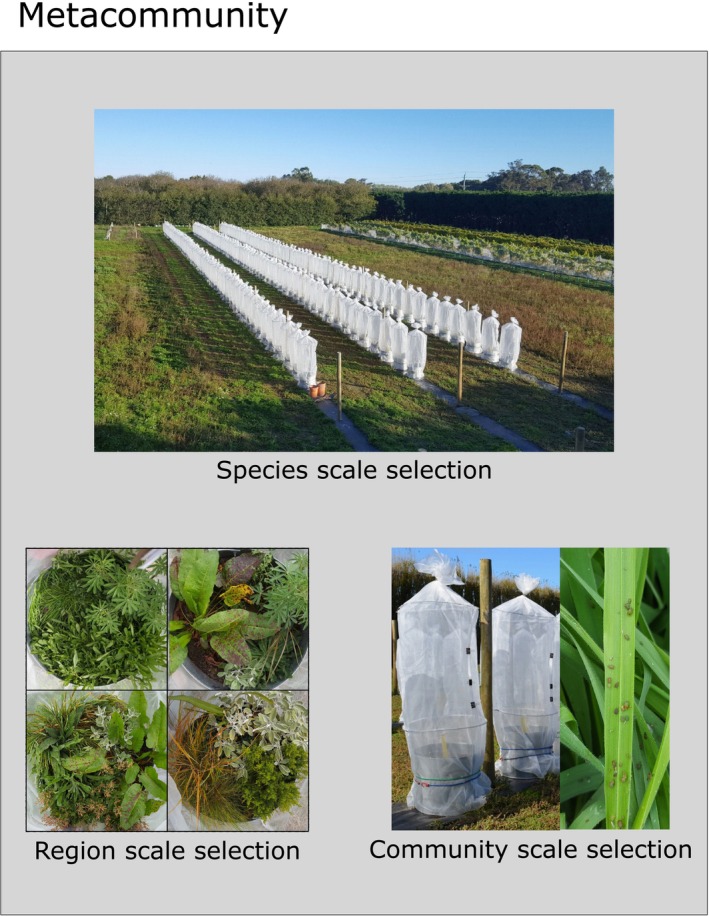
In a biogeographic region, biodiversity change can reflect processes acting at multiple scales. Species‐scale selection occurs when a species increases in relative abundance across the entire biogeographic region; in our case, a series of experimental plant communities with mesh cages used to enclose invertebrate herbivore communities. Habitat‐scale selection occurs when a species increases in abundance in some environments relative to others; in our case, individual mesh enclosures were planted with one of 20 different combinations of host plant species (with plant communities acting as “habitats”; four shown here as an example). Community‐scale selection occurs when a species increases in abundance in some communities relative to others; in our case, a few individual enclosures where an individual insect species increased in abundance (mostly *Rhopalosiphum padi* aphids, shown here on the exotic host grass *Holcus lanatus* ).

To demonstrate the partitioning of selection at different scales, we quantified temporal changes in the Shannon diversity of invertebrate herbivores inhabiting experimental grassland metacommunity (Figure 3, Appendix S2: Figure S1), designed to mimic the biodiversity of grassland communities in New Zealand. This metacommunity consisted of 20 distinct habitats, defined by plant community composition (Appendix S2: Figure S2, Table S2), and which were designed to vary orthogonally in the proportion of exotic and woody species (0%–100% and 0%–63%, respectively). In turn, each habitat was replicated in four distinct mesocosm communities (Appendix S2: Figure S3). Previous papers report the community‐scale outcomes (Waller et al. 2020), plant–herbivore interactions (Allen et al. 2021) and plant–pathogen interactions (Waller et al. 2024) for the same communities. Here we use this dataset to explore how changes in herbivore abundance over time affect alpha, beta, and gamma diversity as Shannon diversity.

A total of 20 invertebrate herbivore species (Appendix S2: Table S3) that varied in provenance, phylogeny, and traits were added to the plant communities (Waller et al. 2020). Thirteen herbivore species successfully established from intentional additions, with the same number of individuals of each species added to each community. Herbivore species additions were carried out over several months of the experiment, depending upon availability of some species (see Supporting Information for detailed protocols for each herbivore species). To establish the herbivore communities, the mesocosms were covered with large mesh cages to keep added herbivores enclosed and deter most naturally occurring external herbivores (Appendix S2: Figure S2; see Supporting Information for detailed description of cages). Because the mesh cages did not entirely deter aphids and some other small invertebrates, seven additional species immigrated into some communities. In keeping with the original experimental design, individuals of commonly self‐introduced species were added to other cages so that they would have the opportunity to establish throughout the entire experiment. Establishment success and other herbivore species characteristics are detailed in Appendix S2: Table S3. The number of herbivores was surveyed on eight occasions: May, June, July, August, September, and November in 2017, and January and April in 2018. Unlike many diversity surveys, the goal was to measure total abundances of all species in the community, rather than a sample of a broader community. In view of this, we did not correct for sampling effort (Roswell et al. 2021).

We computed the change in biodiversity between two consecutive surveys (hereafter one sampling period). We then partitioned this change in biodiversity using Equations (4) and (7) (see figshare repository). To do this, we divided individuals in the present survey into two categories: immigrants to the biogeographic region and residents. In our system, communities were isolated from each other but surrounded by seminatural habitat with abundant insects. Therefore, we assumed that any individual belonging to species previously unobserved in a given community was likely to be an immigrant to the biogeographic region. We assumed that residents were individuals belonging to species that were observed in the community in the previous sampling period. This analysis was performed using scripts developed in R (R Development Core Team 2006; see figshare repository).

To determine whether the partitions we identify include significant information related to the dynamics of the metacommunity, we used a parametric bootstrapping approach to assess the importance of variation in species abundances due to chance. To do this, we assumed that the present counts observed for each species at each community are fixed. We then simulated counts for species *i* in habitat *j* and community *k* in the future using realizations from a Poisson probability distribution with a mean equal to the observed present count of species *i* in location *j*. A total of 1000 simulated datasets were obtained. From these, we estimated empirical 95% confidence intervals for total biodiversity change in alpha, beta, and gamma diversity, along with change due to selection at each scale, transmission, and immigration.

### 3.1 Results

**FIGURE 4:**
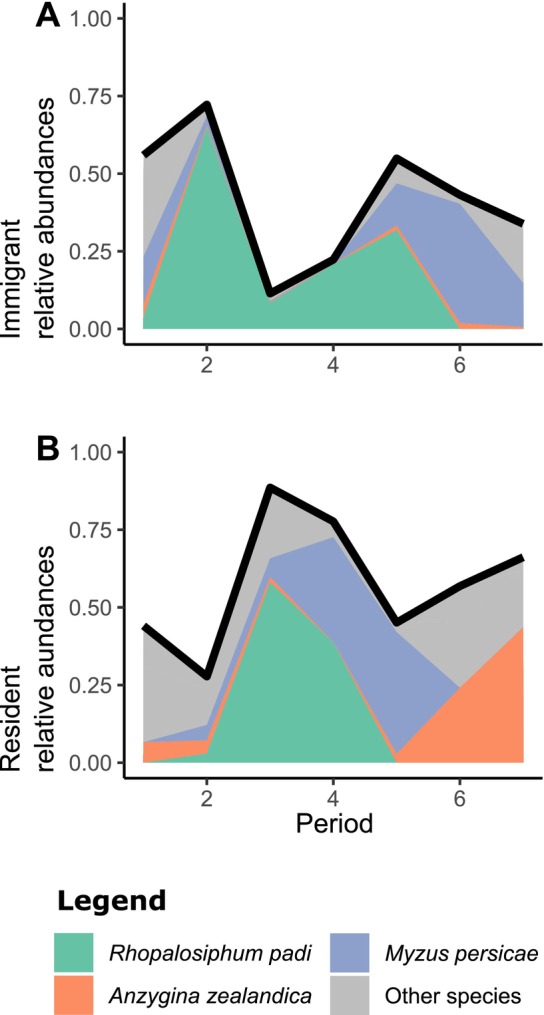
Summary of shifts in relative abundances of species across the biogeographic region. The impact of these shifts on biodiversity are captured by the species‐level selection term. (A) shows the relative abundance of immigrants by period (black line), with the relative abundances of irruptive species highlighted, including *Rhopalosiphum padi* (green), *Anzygina zealandica* (orange), and *Myzus persicae* (blue). Immigrants of species other than these three are colored in gray. (B) Shows relative abundances among residents, with the black line denoting the proportion of resident species (1—the proportion of immigrants).

Across the biogeographic region, aphid outbreaks dominated changes in relative abundances (Figure 4). Some aphid outbreaks were seasonal, occurring in the austral spring of October–November. This suggests that fitness differences in our system are a response to phenology. Notably, at the end of the second sampling period, 65% of individuals were cherry‐oat aphids, *Rhopalosiphum padi* , that had immigrated into new communities (a further 3% were residents in communities where the species had previously been detected). This species then became resident and reproduced prodigiously in the third period (Figure 4B). In sampling periods four and five, individuals of this species were artificially added to new communities, leading to another, smaller wave of immigration. This species declined in subsequent periods. There were also spikes in the abundance of other aphids, such as a wave of immigration of the green peach aphid, *Myzus persicae* , at observation period six.

**FIGURE 5:**
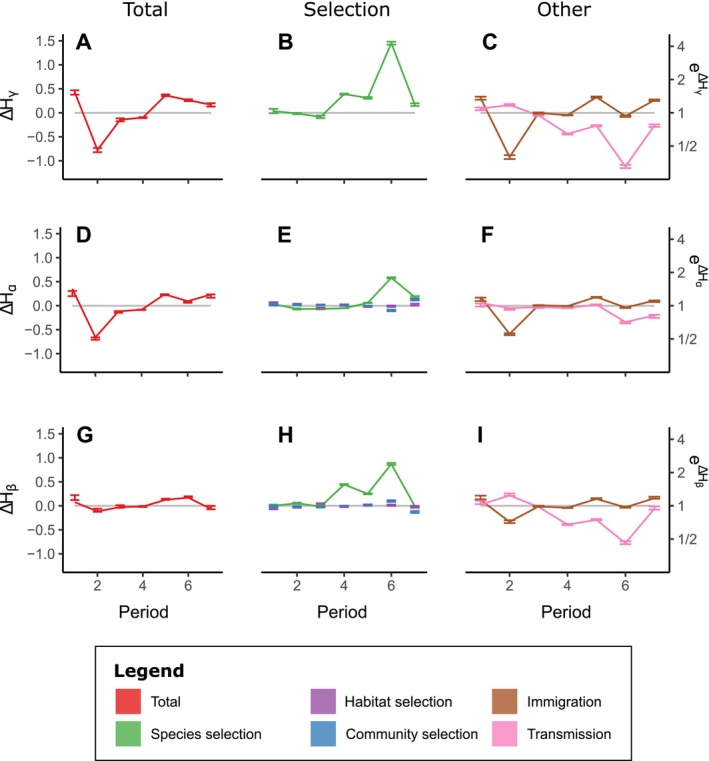
Biodiversity declined during the initial phase and then rebounded gradually for gamma (A), alpha (D), and beta (G) diversity. In general, species‐scale selection (green) explained the rebounds in biodiversity (gamma, B; alpha, E; beta, H), although it had the strongest effect on gamma diversity. Note the horizontal bars denote 95% bootstrapped confidence intervals. Species‐scale selection was partially obscured by transmission (pink; gamma, C; alpha, F; beta, I). Immigration (brown) explained the decline of biodiversity at period 2 (gamma, C; alpha, F; beta, I). Habitat‐scale selection (purple) had negligible effects (alpha, E, beta, H). Community‐scale selection (blue) also had negligible effects (alpha, E, beta, H). Since gamma diversity treats all individuals of a given species equally regardless of their location, it is insensitive to the other two selection terms. The y‐axis is presented in units of change in Shannon entropy ∆H (axis on left hand side of plot). Some researchers find it more intuitive to present analyses of biodiversity using $e^{∆H}$ the equivalent number of uniformly distributed species (i.e., Hill numbers); for this reason, we present this scale on the right‐hand side.

There was a sharp decline in gamma diversity at period two, followed by a milder rebound at period five (Figure 5A). The decline at period two was due to the immigration of new species, particularly of *R. padi* (Figure 4A). This species became so abundant that it decreased the evenness of species relative abundances, reducing gamma diversity. The subsequent increase in Shannon gamma diversity was associated with a bout of selection favoring rare species (Figure 5B; green line). However, the overall change in Shannon gamma diversity was far weaker than the strength of selection. This difference occurred because of transmission (Figure 5C; pink line), which counteracted the effects of selection. The large transmission term reflects the fact that rare species displaced common species.

The total change in gamma diversity (Figure 5A) can be divided into contributions from the total change in alpha diversity (Figure 5D) and total change in beta diversity (Figure 5G). Much of the change in gamma diversity was due to changes in alpha diversity across the experiment (Figure 5D). In particular, species‐scale selection increased gamma diversity substantially in period six (Figure 5E). Immigration of *R. padi* decreased gamma diversity at period two and transmission counteracted selection at period five (Figure 5F). Though the experiment was designed to study habitat‐scale differences (i.e., differences in plant communities), selection was weak at both the habitat and community scale, explaining only a mild decline in alpha diversity at period six (Figure 5E, purple and blue lines).

Changes in beta diversity were smaller than those in alpha diversity, but they followed a similar trajectory (Figure 5G). Once again, selection among species peaked at period six (Figure 5H). Immigration again decreased biodiversity at period two and transmission counteracted species‐scale selection.

## 4 Discussion

Though much of the Earth's diversity is structured hierarchically, it is difficult to separate mechanisms shaping biodiversity at each scale. In response to this challenge, we have proposed a novel application of selection theory. When applied to a large‐scale community dataset, our results showed small effects of selection among habitats, a surprising result given that the experiment was designed to study habitat‐scale differences. Instead, diversity responded strongly to immigration of new aphid species, with strong effects of species‐scale selection obscured by a strong transmission effect, which resulted from the replacement of common species with rare species. Below we highlight the value of distinguishing mechanisms acting on diversity at multiple scales.

In our example dataset, partitioning helped to identify the consequences of rapid shifts in species relative abundances. The species‐scale selection term was strong, indicating rapid shifts in species' overall relative abundances across the metacommunity. This result seems to be a consequence of seasonal outbreaks of aphids, irrespective of plant community composition or mesocosm community. Relative to species‐scale selection, selection at the habitat and community scales were weak (Figure 5). This is unexpected because the experiment was designed to highlight differences among the 20 types of plant communities (i.e., habitat‐scale selection). This result likely indicates that aphids' success was only weakly determined by the plant community. Furthermore, selection reached a peak in sampling period six, but with no corresponding increase in diversity. The reason for this was that the large shifts in relative abundance tended to replace common species with previously rare species—a phenomenon captured by the transmission term (Figure 5). Note that this effect of transmission would not have been obvious in previous work such as Godsoe et al. (2022), which measured species‐scale selection but merged lower‐scale selection terms with transmission. The current dataset emphasizes the need to quantify selection at different spatial scales, since a simpler partitioning scheme can merge selection with transmission. Another major surprise was that immigration of aphids in period two drove a decrease in biodiversity.

Partitioning was particularly useful in our case because the dominant mechanism of diversity change (aphid outbreaks) produced unexpected consequences. Rapid growth in aphids can have surprisingly strong effects on experiments concerning plant–insect interactions (Agrawal et al. 2005). Aphid population growth is determined by multiple interconnected explanations (Dixon 1977). Once established in mid‐summer, aphids reproduce rapidly, with many species such as *R. padi* deriving a short‐term fitness advantage by producing wingless asexual offspring (Dixon 1977). Rapid declines in these species may be a result of natural enemies such as predators and parasitoids (Dixon 1977). The mesh enclosures in our experiment did reduce the effects of natural enemies, although small numbers of lacewings, parasitoids, and ladybeetles were observed in some communities. Another potential explanation for these declines is the changes in plants caused by aphids, including induced defenses and lower nutritional value (Liu et al. 2020). Declines may also be due to phenological shifts in aphids, including a late‐season return to sexual reproduction. Filtering based on the abiotic environment is unlikely to be important given that all of the communities were near each other, and treatments were randomized. The advantage of our approach is that it focuses on differences in fitness, which are far easier to quantify than the consequences of interactions among species.

By focusing on selection, we were able to logically separate changes in relative abundances operating at different scales. Probabilities at one scale can be re‐expressed as a product of conditional probabilities across scales (Equation 1). This fact makes it possible to study sources of changes in relative abundances at each scale. It is far more difficult to separate the effects of species interactions such as competition at broad spatial scales. For example, the analysis of competition in modern coexistence theory tends to assume that spatial variation in fitness is weak (Ruel et al. 1999; Chesson et al. 2005; Denny 2017), an approximation that is unlikely to hold in nature (Ellner et al. 2019). Similarly, effects of biotic interactions and the abiotic environment are often conflated (Cadotte and Tucker 2017; Barner et al. 2018; Thurman et al. 2019; Poggiato et al. 2021). A recent meta‐analysis of how assembly processes shaped beta diversity investigated spatial and environmental covariates, but could only speculate on the role of fitness (Nishizawa et al. 2022). In contrast our approach quantifies fitness differences, and their effects on diversity.

Our analysis of the effects of selection is easier to implement than tests of environmental filtering (Kraft et al. 2015). To apply our approach requires datasets that contain simultaneous measures of relative abundances at multiple scales, repeated over multiple time points. These criteria are met by many available datasets (Dornelas et al. 2018). Partitioning approaches such as ours may be applied across timescales ranging from hours (Collins and Gardner 2009) to millennia (Rankin et al. 2015). However, the dominant drivers of diversity change over short time intervals will likely differ from answers over longer time intervals. In contrast, it can be extremely difficult to tease apart the effects of competition on diversity from observational data alone (Connor and Simberloff 1979; Kraft et al. 2015; Cadotte and Tucker 2017; Blanchet et al. 2020; Godsoe et al. 2023).

Immigrants play a distinctive role in the mechanisms that shape biodiversity (Cadotte and Fukami 2005). For example, dispersal often interacts with competition to afford novel avenues for the maintenance of biodiversity (Berkley et al. 2010). Unfortunately, it is very difficult to separate the effects of immigration from competition even in theory (Abrams and Wilson 2004; Snyder and Chesson 2004; Snyder et al. 2005; Berkley et al. 2010). An advantage of our approach is that there is a logical distinction between the effects of selection and the effects of dispersal (Kerr and Godfrey‐Smith 2009). In theory, this makes it easy to quantify the amount of change due to immigration, by separating immigrants from residents. In practice, of course, there will still be uncertainty in whether a given individual is an immigrant or not. In some cases, this distinction will be evident, such as the self‐introduction of aphids in our experiment. However, in our experiment it is only practical to detect the immigration of new species into a community. There is always a risk that individuals counted as residents in fact represent immigrants that have been misclassified. This would leave the overall effect of aphids on biodiversity unchanged but would underestimate the impact of selection and overestimate the impact of transmission. Therefore our approach relaxes the assumptions that we need to make about the role of immigration, and lessens the need for complex dynamic models such as those in (Abrams and Wilson 2004, Snyder and Chesson 2004, Snyder et al. 2005, Berkley et al. 2010). At the same time, we have illustrated our approach with relatively simple assumptions about immigration in our empirical study (i.e., that immigrants belong to species that are new to a community and all other individuals are residents). When more data are available on the strength of immigration in an experiment, further work could be done to probe this assumption. For example, one might assume a distribution for the proportion of immigrants during each sampling period and assess uncertainty via Monte Carlo methods. This is worth further investigation but lies outside the scope of the current study.

To measure immigration (Figure 2B) our model distinguishes new immigrants to the biogeographic region from residents already present in the biogeographic region. Consistent with previous literature on selection, an immigrant may be an individual that arrived between the “present” and “future” observation periods, or a descendant of an individual that arrived between the “present” and “future” observation periods (Frank 2012a). This is important because some species such as aphids can have an extremely short lifespan, and as such we expect that the community included “second generation” immigrants, that is, individuals found in the future community whose parents immigrated into the community. Note that this is a measure of overall immigration into the biogeographic region, though with a great deal of notation it would be possible to further distinguish immigrants from one habitat to another, or one community to another (Rice 2004; Kerr and Godfrey‐Smith 2009; Frank 2012a).

The mechanism of ecological drift is unlikely to drive the diversity changes that we observed in our example dataset. Drift is strongest when population sizes are small and species have similar population growth rates (Hubbell 2001; Vellend 2016; Siqueira et al. 2020). Neither condition was met in our dataset, as we observed rapid shifts in abundance in communities of tens of thousands of individuals. Therefore, as a simplification, we do not formally model drift's effects. Exact predictions for drift can be derived from a null model, assuming all species have (on average) the same fitness. An example is provided in Godsoe et al. (2022) showing that some of the changes in diversity are consistent with drift in a long‐term analysis of vegetation plot data. This is a system where many tree species have similar population growth rates.

Our work demonstrates how analyses of biodiversity change across scales can be connected to observations of fitness. Though it has long been hoped that analyses of biodiversity will clarify the mechanisms that mediate community assembly (Whittaker 1965), our approach highlights an unexpected disconnect when biodiversity change is rapid. The strength of this disconnect can be measured using the transmission term we describe in (Equation 7). This term is high when rare species rapidly shift to become common, resulting in a negligible change in diversity despite high turnover of species dominance. Using empirical data, we highlight the importance of this mechanism relative to selection in shaping biodiversity change across scales. This work suggests that improved forecasts of biodiversity changes may require a nuanced understanding of how shifts in relative abundances across a biogeographic region translate into shifts in diversity.

## Author Contributions

**William Godsoe:** conceptualization (equal), formal analysis (equal), writing – original draft (lead), writing – review and editing (lead). **Warwick J. Allen:** data curation (equal), methodology (equal), writing – original draft (supporting), writing – review and editing (supporting). **Lauren P. Waller:** data curation (equal), methodology (equal), project administration (equal), writing – original draft (supporting), writing – review and editing (supporting). **Barbara I. P. Barratt:** conceptualization (equal), data curation (equal), formal analysis (equal), writing – review and editing (supporting). **Sarah P. Flanagan:** conceptualization (supporting), writing – original draft (supporting), writing – review and editing (supporting). **Zachary H. Marion:** investigation (supporting), writing – original draft (supporting), writing – review and editing (supporting). **Jason M. Tylianakis:** project administration (equal), resources (equal), supervision (equal), writing – original draft (supporting), writing – review and editing (supporting). **Elena Moltchanova:** formal analysis (equal), software (equal), writing – original draft (supporting), writing – review and editing (supporting). **Ian A. Dickie:** funding acquisition (equal), investigation (equal), project administration (equal), supervision (equal), writing – original draft (supporting), writing – review and editing (supporting).

## Conflicts of Interest

The authors declare no conflicts of interest.

## Supporting information


**Appendix S1.** Mathematical derivations.

**Appendix S2.** Experiment details.

## Data Availability

Code and example dataset associated with this paper are provided in an online data repository: https://doi.org/10.6084/m9.figshare.c.7829201.v1.
