# Supplementary material for: Rapid Shifts in Relative Abundance Obscure Temporal Diversity Changes in a Metacommunity
Source: Ecol Evol. 2025 Jul 2;15(7):e71694. doi: 10.1002/ece3.71694 (PMC12222622; doi:10.1002/ece3.71694)
Supplement: Supplementary file 1 — Appendix S1. Mathematical derivations. [file ECE3-15-e71694-s002.pdf]

Partition in the presence of immigration

From Equation 1 in the main text, Shannon entropy diversity indices can be written as:

$$H_\circ = \sum_i \sum_j \sum_k p_{ijk} z_{ijk\circ}. \tag{1}$$

Here p_{ijk} is the proportion of individuals in species i, habitat j and community k. The contribution that a given individual makes to diversity is equal to $z_{ijk\circ}$. The $\circ$ specifies the diversity component (either α, β, or γ). See Table S1 for definitions.

A similar formula can be used to calculate Shannon entropy in the future observation period:

$$H'_\circ = \sum_i \sum_j \sum_k \pi'_{ijk} z'_{ijk\circ}. \tag{2}$$

Here we denote the relative abundances with a π rather than a p. The individual contributions to diversity $z'_{ijk\circ}$, likewise use the relative abundances in the future time period.

To understand the mechanisms changing diversity we separate Equation 2 out into contributions of immigrants and residents:

$$H'_\circ = \varphi H'_{\circ,I} + (1-\varphi) H'_{\circ,D}. \tag{3}$$

This formula requires careful book keeping and so we define all the relevant terms starting with absolute abundances ν' in the future observation period. Some of these individuals are divided into immigrants (m') to the metacommunity, which have arrived since the initial sampling period. All other individuals are residents, whose abundance are denoted (n'). This leads to the following updated expression for the total abundance of species i in habitat j and community k.

$$\nu'_{ijk} = n'_{ijk} + m'_{ijk}.$$

The proportion of all individuals in the metacommunity that are immigrants is given by:

$$\varphi = \frac{n'_{\bullet\bullet\bullet}}{n'_{\bullet\bullet\bullet} + m'_{\bullet\bullet\bullet}}$$

The proportion of individuals that are residents is given by $1-\varphi$.

The diversity of immigrants to the metacommunity is then an average of the contributions across all immigrants. This is equal to:

$H'_{\circ,I} = \sum_i \sum_j \sum_k q'_{ijk} z'_{ijk\circ}$.

Here q'_{ijk} is the proportion of immigrants i habitat j and community k:

$$q'_{ijk} = \frac{m'_{ijk}}{m'_{\bullet\bullet\bullet}}$$

Similarly the diversity of residents in the metacommunity is an average of the contributions across all residents. This is equal to:

$$H'_{\circ,D} = \sum_i \sum_j \sum_k p'_{ijk} z'_{ijk\circ} \tag{4}$$

where p'_{ijk} is the proportion of residents i habitat j and community k:

$$p'_{ijk} = \frac{n'_{ijk}}{n'_{\bullet\bullet\bullet}}.$$

Change in diversity

The change in diversity over time is given by:

$$\Delta H_\circ = H'_\circ - H_\circ$$

Substituting in Equation 3 gives:

$$= \varphi H'_{\circ,I} + (1-\varphi) H'_{\circ,D} - H_\circ \tag{5}$$

To facilitate comparisons between $H'_{\circ,D}$ and $H_\circ$, we re-write the formula for the initial diversity as $H_\circ = \varphi H_\circ + (1-\varphi) H_\circ$. We then then factor Equation 5 giving:

$$= \varphi \underbrace{\left(H'_{\circ,I} - H_\circ\right)}_{immigrants} + (1-\varphi) \underbrace{\left(H'_{\circ,D} - H_\circ\right)}_{residents} \tag{6}$$

This is Equation 4 in the main text.

Changes in relative abundance

Next we dig into how fitness differences at different spatial scales lead to differences between the initial observation of the community and the final observation of the community. Starting from 5 we have:

$$H'_{\circ,D} - H_\circ$$

To explicitly label individual contributions we substitute in Equations 1 and 4:

$$= \sum_i \sum_j \sum_k p'_{ijk} z'_{ijk\circ} - \sum_i \sum_j \sum_k p_{ijk} z_{ijk\circ}$$

In this equation, the relative abundances p_{ijk} aggregate information across all spatial scales. To tease apart contributions from each scale we use the textbook definition of conditional probability to obtain:

$$p_{ijk} = p_{i\bullet\bullet} p_{j|i\bullet} p_{k|ij} = \frac{n_{i\bullet\bullet}}{n_{\bullet\bullet\bullet}} \frac{n_{ij\bullet}}{n_{i\bullet\bullet}} \frac{n_{ijk}}{n_{ij\bullet}}$$

This states that p_{ijk} can be decomposed into the product of probabilities at different scales: the probability that an individual belongs to species i ($p_{i\bullet\bullet}$), the probability that an individual is in habitat j given that it is in species i ($p_{j|i\bullet}$), and the probability that an individual is in community k given that it is in species i and habitat j ($p_{k|ij}$). The same argument applies to p'_{ijk}, these substitutions lead to:

$$= \sum_i \sum_j \sum_k p'_{i\bullet\bullet} p'_{j|i\bullet} p'_{k|ij} z'_{ijk\circ} - \sum_i \sum_j \sum_k p_{i\bullet\bullet} p_{j|i\bullet} p_{k|ij} z_{ijk\circ}$$

An equivalent way to write this is as:

$$= \sum_i p'_{i\bullet\bullet} \sum_j p'_{j|i\bullet} \sum_k p'_{k|ij} z'_{ijk\circ} - \sum_i p_{i\bullet\bullet} \sum_j p_{j|i\bullet} \sum_k p_{k|ij} z_{ijk\circ}$$

The consequences of selection at different levels can then be found by calculating the difference between the contributions that residents make to diversity (in the future observation) and the present diversity:

$$H'_{\circ,D} - H_\circ = \sum_i p'_{i\bullet\bullet} \sum_j p'_{j|i\bullet} \sum_k p'_{k|ij} z'_{ijk\circ} - \sum_i p_{i\bullet\bullet} \sum_j p_{j|i\bullet} \sum_k p_{k|ij} z_{ijk\circ} \tag{7}$$

To partition this equation we take Equation 7, then add the following terms each of which adds to zero:

$$\sum_i p'_{i\bullet\bullet} \sum_j p_{j|i\bullet} \sum_k p_{k|ij} z_{ijk\circ} - \sum_i p'_{i\bullet\bullet} \sum_j p_{j|i\bullet} \sum_k p_{k|ij} z_{ijk\circ} = 0,$$

$$\sum_i p'_{i\bullet\bullet} \sum_j p'_{j|i\bullet} \sum_k p_{k|ij} z_{ijk\circ} - \sum_i p'_{i\bullet\bullet\bullet} \sum_j p'_{j|i\bullet} \sum_k p_{k|ij} z_{ijk\circ} = 0,$$

$$\sum_i p'_{i\bullet\bullet} \sum_j p'_{j|i\bullet} \sum_k p'_{k|ij} z_{ijk\circ} - \sum_i p'_{i\bullet\bullet} \sum_j p'_{j|i\bullet} \sum_k p'_{k|ij} z_{ijk\circ} = 0.$$

This results in the following expression for diversity change:

$$
\begin{aligned}
H'_{\circ,D} - H_{\circ} = & \sum_i p'_{i\bullet\bullet} \sum_j p_{j|i\bullet} \sum_k p_{k|ij} z_{ijk\circ} - \sum_i p_{i\bullet\bullet} \sum_j p_{j|i\bullet} \sum_k p_{k|ij} z_{ijk\circ} \\
& + \sum_i p'_{i\bullet\bullet} \sum_j p'_{j|i\bullet} \sum_k p_{k|ij} z_{ijk\circ} - \sum_i p'_{i\bullet\bullet} \sum_j p_{j|i\bullet} \sum_k p_{k|ij} z_{ijk\circ} \\
& + \sum_i p'_{i\bullet\bullet} \sum_j p'_{j|i} \sum_k p'_{k|ij} z_{ijk\circ} - \sum_i p'_{i\bullet\bullet} \sum_j p'_{j|i} \sum_k p_{k|ij} z_{ijk\circ} \\
& + \sum_i p'_{i\bullet\bullet} \sum_j p'_{j|i} \sum_k p'_{k|ij} z'_{ijk\circ} - \sum_i p'_{i\bullet\bullet} \sum_j p'_{j|i} \sum_k p'_{k|ij} z_{ijk\circ}
\end{aligned}
\tag{8}
$$

This can be simplified by denoting differences between present and future of the same term with Δ:

$$
\begin{aligned}
H'_{\circ,D} - H_{\circ} = & \sum_i \Delta p_{i\bullet\bullet} \sum_j p_{j|i\bullet} \sum_k p_{k|ij} z_{ijk\circ} \\
& + \sum_i p'_{i\bullet\bullet} \sum_j \Delta p_{j|i\bullet} \sum_k p_{k|ij} z_{ijk\circ} \\
& + \sum_i p'_{i\bullet\bullet} \sum_j p'_{j|i\bullet} \sum_k \Delta p_{k|ij} z_{ijk\circ} \\
& + \sum_i p'_{i\bullet\bullet} \sum_j p'_{j|i\bullet} \sum_k p'_{k|ij} \Delta z_{ijk\circ}
\end{aligned}
\tag{9}
$$

This is Equation 7 in the main text.

To derive the full partition of diversity change substitute Equation 9 back into Equation 6 giving:

$$
\begin{aligned}
\Delta H_{\circ} = & \varphi \left(H'_{\circ,I} - H_{\circ} \right) + \\
& (1-\varphi)(\sum_i \Delta p_{i\bullet\bullet} \sum_j p_{j|i\bullet} \sum_k p_{k|ij} z_{ijk\circ}) + \\
& (1-\varphi)(\sum_i p'_{i\bullet\bullet} \sum_j \Delta p_{j|i\bullet} \sum_k p_{k|ij} z_{ijk\circ}) + \\
& (1-\varphi)(\sum_i p'_{i\bullet\bullet} \sum_j p'_{j|i\bullet} \sum_k \Delta p_{k|ij} z_{ijk\circ}) + \\
& (1-\varphi)(\sum_i p'_{i\bullet\bullet} \sum_j p'_{j|i\bullet} \sum_k p'_{k|ij} \Delta z_{ijk\circ})
\end{aligned}
\tag{10}
$$

This is the full partitioning of diversity change, Equation 3 in the main text with the diversity change due to immigration quantified by the term $\varphi(H'_{\circ,I} - H_{\circ})$, species level selection quantified by $(1-\varphi)(\sum_i \Delta p_{i\bullet\bullet} \sum_j p_{j|i\bullet} \sum_k p_{k|ij} z_{ijk\circ})$. Habitat level selection quantified by $(1-\varphi)(\sum_i p'_{i\bullet\bullet} \sum_j \Delta p_{j|i\bullet} \sum_k p_{k|ij} z_{ijk\circ})$. Community level selection is quantified by $(1-\varphi)(\sum_i p'_{i\bullet\bullet} \sum_j p'_{j|i\bullet} \sum_k \Delta p_{k|ij} z_{ijk\circ})$ and transmission bias quantified by $(1-\varphi)(\sum_i p'_{i\bullet\bullet} \sum_j p'_{j|i\bullet} \sum_k p'_{k|ij} \Delta z_{ijk\circ})$

Conversion from entropy to "true diversities"

Shannon entropy is expressed in units of uncertainty or "suprize" that are unfamiliar to many ecologists (Cover and Thomas 2005). Godsoe et al. (2022) describes how partitions of Shannon entropy can be converted into partitions of "true diversities" (Jost 2006, Jost 2007), with units that are arguably more intuitive. α and γ diversity are reported in the number uniformly distributed species required to produce the observed measurement of Shannon entropy, while β diversity is reported in the number of uniform communities needed to produce the observed measurement of Shannon entropy.

Exponentiation converts Shannon entropy to its numbers equivalent (i.e. $e^{H_\circ}$). We apply the same technique to study the relationship between mechanisms and diversity change.

We start with Equation 10, but to improve readability we replace the terms for S_1 for species level selection, S_2 for treatment level selection, S_3 for community level selection, T for change in $z_{ijk\circ}$ and I for immigration giving:

$$\Delta H_\circ = S_{1\circ} + S_{2\circ} + S_{3\circ} + T_\circ + I_\circ \tag{11}$$

Exponentiation of Equation 11 then gives:

$$\frac{e^{H'_\circ}}{e^{H_\circ}} = e^{S_{1\circ}} e^{S_{2\circ}} e^{S_{3\circ}} e^{T\circ} e^{I\circ} \tag{12}$$

In words, the left hand side of Equation 12 describes change in diversity as the ratio of true diversity in the future to true diversity in the present observations. This ratio is equal to multiplicative effects of each of the mechanisms, converted into units of numbers equivalents. Recall that by the quotient rule for exponents:

$e^{\Delta H_\circ} = \frac{e^{H'_\circ}}{e^{H_\circ}}$.

Figure 5 in the main text simultaneously presents results using two sets of units, the first is based on change in Shannon entropy ($\Delta H_\circ$) as presented in Equation 10. Since the terms in Equation 12 are just the terms in Equation 11 exponentiated, we can display them on Figure 5 simply by adjusting the axis. Note however that these terms have multiplicative effects on diversity, while the partition in the main text has additive effects. Also, the confidence intervals we provide are calculated for the entropy not its numbers equivalent.

Table S1. List of terms.

Term	Definition
$\bullet$	Indicates summing across an index, such that $n_{\bullet\bullet\bullet} = \sum_i \sum_j \sum_k = n_{ijk}$.
$\circ$	Indicates the diversity component of interest (one of α, β or γ)
Δ	Change in a variable between future and present
$H_\circ$, $H'_\circ$	A Shannon entropy diversity index, in the present and future respectively.
$H'_{\circ,I}$, $H'_{\circ,D}$	Contributions to future diversity of (respectively) immigrants and residents.
i, j, k	Indexes for species, habitats and communities, respectively.
ν', n', m'	In the future observation period, the absolute abundance of respectively all individuals, residents and immigrants.
p_{ijk}	The probability that an individual in the present observation period belongs to species i, habitat j and community k.
$p_{i\bullet\bullet}$	The probability that an individual in the present observation period belongs to species i, across the metacommunity. Equivalently the relative abundance of species i across the metacommunity.
$p_{i\|jk}$	The probability that in the present observation period an individual belongs to species i given that it is found in community k of habitat j. This is equivalent to the relative abundance of species i in community k of habitat j.
$p_{j\|i\bullet}$	The probability that in the present observation period an individual belongs to species i given that it is found in habitat j.
$p_{k\|ij}$	The probability that in the present observation period, an individual belongs to community k given that it is in species i and habitat j.
π', p', q'	Refers to probabilities in the future observation period for respectively, 1) any individual, 2) residents or 3) immigrants.
$z_{ijk\circ}$, $z'_{ijk\circ}$	Individual contributions to diversity in the present and future observation periods respectively.
φ	Proportion of individuals in the future observation period that are immigrants.
