# Supplementary material for: Rapid Shifts in Relative Abundance Obscure Temporal Diversity Changes in a Metacommunity
Source: Ecol Evol. 2025 Jul 2;15(7):e71694. doi: 10.1002/ece3.71694 (PMC12222622; doi:10.1002/ece3.71694)
Supplement: Supplementary file 2 — Appendix S2. Experiment details. [file ECE3-15-e71694-s001.docx]

**Supplementary Information for Godsoe et al.,** **A multilevel mechanistic model of diversity change**

**Supplementary Figures**

**
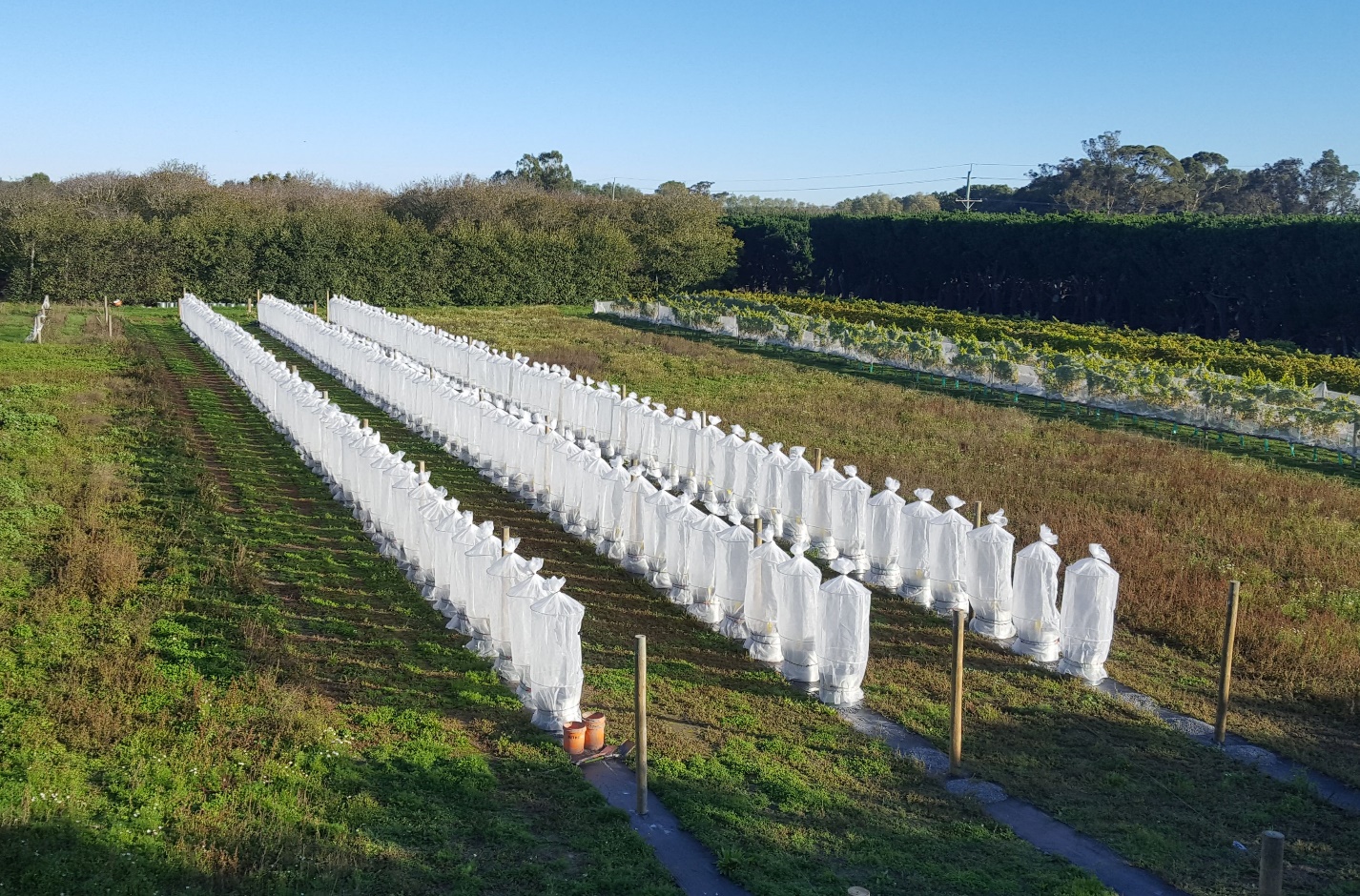
**

**Figure S1. Experimental mesocosm communities in the field.** Communities are shown immediately after planting.

**
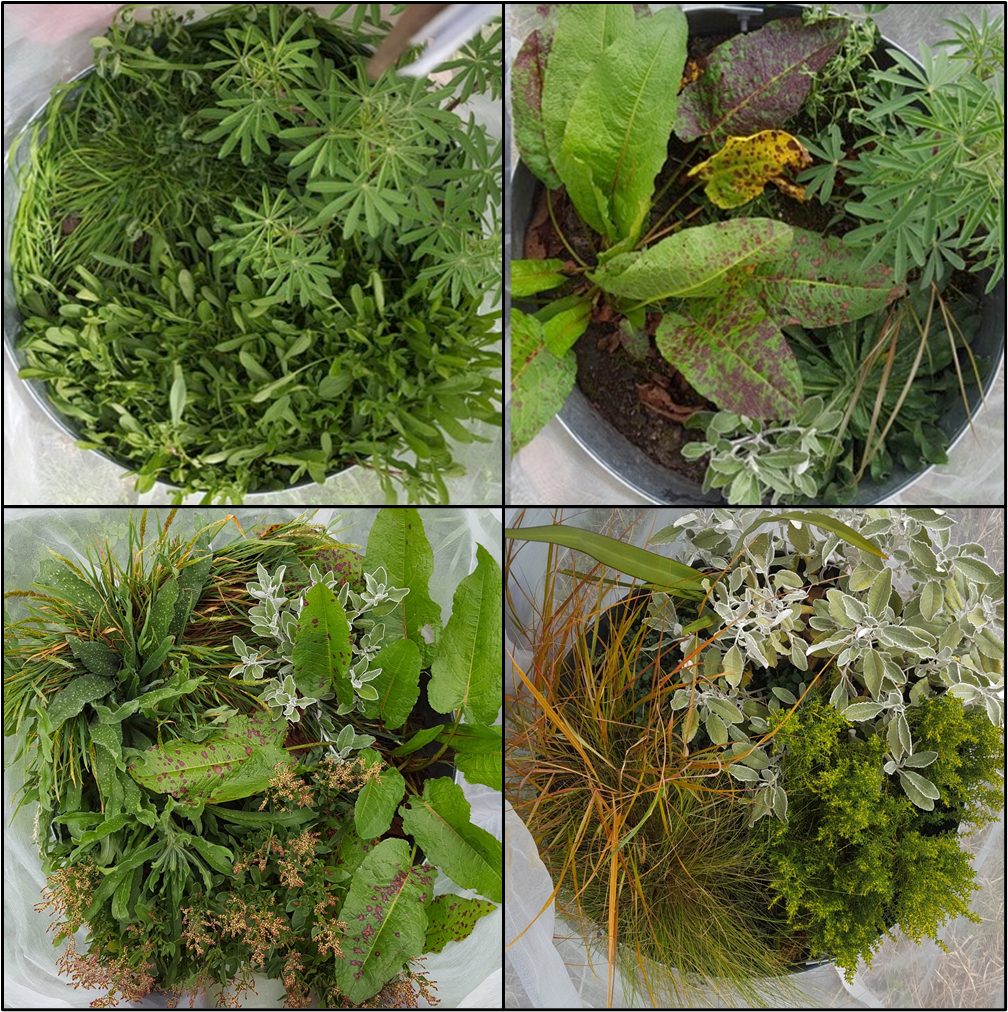
**

**Figure S2. Experimental mesocosm communities.** Example experimental mesocosm communities after eight months of growth (community number, clockwise from top left: 16, 12, 5, 3).


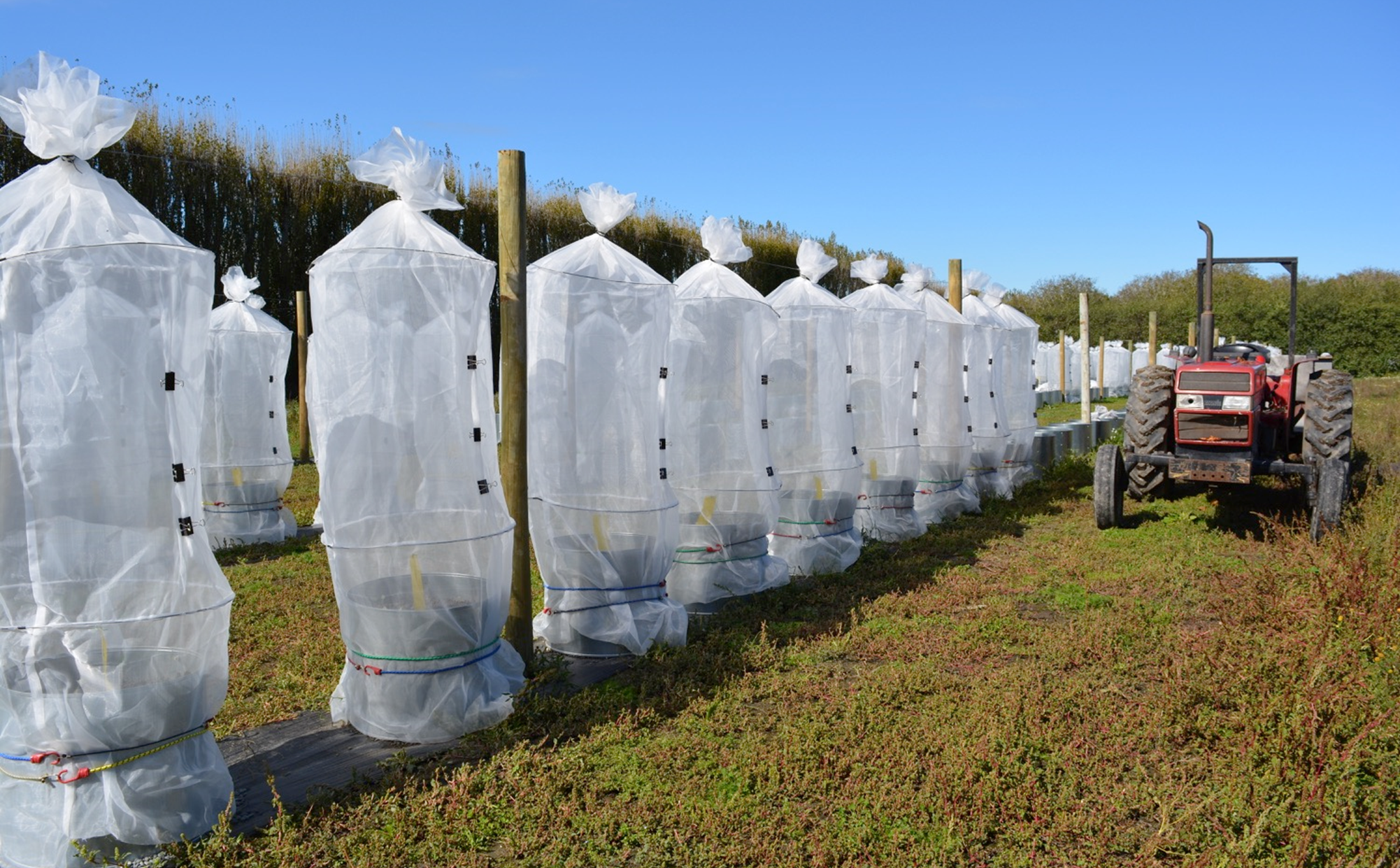


**Figure S3. Photo of herbivore cages.** Herbivore cages installed on mesocosm pots prior to planting.

**Supplementary Tables**

**Table S1.** Plant species composition and proportion of exotic (P.e.) and woody (P.w.) plants of each mesocosm community.

| Community | Plant species | Community | Plant species | Community | | Plant species |
| --- | --- | --- | --- | --- | --- | --- |
| 1 | *Agrostis capillaris* | 2 | *Achillea millefolium* | 3 | | *Acaena inermis* |
| P.e. = 1.00 | *Anthoxanthum odoratum* | P.e. = 0.25 | *Cirsium vulgare* | P.e. = 0.50 | | *Anthoxanthum odoratum* |
| P.w. = 0.00 | *Holcus lanatus* | P.w. = 0.00 | *Dactylis glomerata* | P.w. = 0.00 | | *Brachyglottis greyi* |
|  | *Hypericum perforatum* |  | *Echium vulgare* |  | | *Chionochloa conspicua* |
|  | *Lolium perenne* |  | *Festuca novae-zelandiae* |  | | *Echium vulgare* |
|  | *Rumex obtusifolius* |  | *Medicago sativa* |  | | *Poa colensoi* |
|  | *Trifolium pratense* |  | *Ozothamnus leptophyllus* |  | | *Rumex acetosella* |
|  | *Trifolium repens* |  | *Rumex acetosella* |  | | *Rumex obtusifolius* |
| 4 | *Acaena caesiiglauca* | 5 | *Acaena caesiiglauca* | 6 | | *Achillea millefolium* |
| P.e. = 0.75 | *Anemanthele lessoniana* | P.e. = 1.00 | *Acaena inermis* | P.e. = 0.00 | | *Cirsium vulgare* |
| P.w. = 0.00 | *Carex secta* | P.w. = 0.00 | *Anemanthele lessoniana* | P.w. = 0.25 | | *Holcus lanatus* |
|  | *Festuca novae-zelandiae* |  | *Brachyglottis greyi* |  | | *Lolium perenne* |
|  | *Hypericum perforatum* |  | *Festuca novae-zelandiae* |  | | *Lupinus arboreus* |
|  | *Medicago sativa* |  | *Ozothamnus leptophyllus* |  | | *Pinus contorta* |
|  | *Phormium cookianum* |  | *Phormium cookianum* |  | | *Trifolium pratense* |
|  | *Poa cita* |  | *Poa colensoi* |  | | *Trifolium repens* |
| 7 | *Achillea millefolium* | 8 | *Acacia dealbata* | 9 | | *Anemanthele lessoniana* |
| P.e. = 0.25 | *Agrostis capillaris* | P.e. = 0.50 | *Acaena caesiiglauca* | P.e. = 0.75 | | *Anthoxanthum odoratum* |
| P.w. = 0.25 | *Carex secta* | P.w. = 0.25 | *Acaena inermis* | P.w. = 0.25 | | *Brachyglottis greyi* |
|  | *Dactylis glomerata* |  | *Alnus glutinosa* |  | | *Carex secta* |
|  | *Hypericum perforatum* |  | *Cirsium vulgare* |  | | *Coprosma robusta* |
|  | *Ozothamnus leptophyllus* |  | *Phormium cookianum* |  | | *Festuca novae-zelandiae* |
|  | *Pinus radiata* |  | *Poa cita* |  | | *Muehlenbeckia astonii* |
|  | *Ulex europaeus* |  | *Trifolium pratense* |  | | *Rumex acetosella* |
| 10 | *Acaena caesiiglauca* | 11 | *Acacia dealbata* | 12 | | *Alnus glutinosa* |
| P.e. = 1.00 | *Carex secta* | P.e. = 0.00 | *Agrostis capillaris* | P.e. = 0.25 | | *Anemanthele lessoniana* |
| P.w. = 0.25 | *Festuca novae-zelandiae* | P.w. = 0.38 | *Dactylis glomerata* | P.w. = 0.38 | | *Brachyglottis greyi* |
|  | *Leptospermum scoparium* |  | *Holcus lanatus* |  | | *Echium vulgare* |
|  | *Olearia virgata* |  | *Lolium perenne* |  | | *Lupinus arboreus* |
|  | *Ozothamnus leptophyllus* |  | *Pinus radiata* |  | | *Medicago sativa* |
|  | *Phormium cookianum* |  | *Trifolium repens* |  | | *Pinus contorta* |
|  | *Poa cita* |  | *Ulex europaeus* |  | | *Rumex obtusifolius* |
| 13 | *Acacia dealbata* | 14 | *Acaena inermis* | 15 | | *Acaena inermis* |
| P.e. = 0.50 | *Achillea millefolium* | P.e. = 0.75 | *Anemanthele lessoniana* | P.e. = 1.00 | | *Carex secta* |
| P.w. = 0.38 | *Carex secta* | P.w. = 0.38 | *Echium vulgare* | P.w. = 0.38 | | *Leptospermum scoparium* |
|  | *Hypericum perforatum* |  | *Holcus lanatus* |  | | *Ozothamnus leptophyllus* |
|  | *Leptospermum scoparium* |  | *Muehlenbeckia complexa* |  | | *Phormium cookianum* |
|  | *Poa colensoi* |  | *Poa cita* |  | | *Poa cita* |
|  | *Rumex obtusifolius* |  | *Podocarpus totara* |  | | *Sophora microphylla* |
|  | *Hebe odora* |  | *Sophora microphylla* |  | | *Hebe odora* |
| 16 | *Agrostis capillaris* | 17 | *Anthoxanthum odoratum* | 18 | | *Alnus glutinosa* |
| P.e. = 0.00 | *Alnus glutinosa* | P.e. = 0.25 | *Coprosma robusta* | P.e. = 0.5 | | *Brachyglottis greyi* |
| P.w. = 0.63 | *Lupinus arboreus* | P.w. = 0.63 | *Lolium perenne* | P.w. = 0.63 | | *Cirsium vulgare* |
|  | *Pinus contorta* |  | *Lupinus arboreus* |  | | *Lupinus arboreus* |
|  | *Pinus radiata* |  | *Ozothamnus leptophyllus* |  | | *Muehlenbeckia complexa* |
|  | *Rumex acetosella* |  | *Pinus contorta* |  | | *Olearia virgata* |
|  | *Trifolium pratense* |  | *Pinus radiata* |  | | *Phormium cookianum* |
|  | *Ulex europaeus* |  | *Ulex europaeus* |  | | *Ulex europaeus* |
| 19 | *Acaena caesiiglauca* | 20 | *Carex secta* |  | |  |
| P.e. = 0.75 | *Muehlenbeckia astonii* | P.e. = 1.00 | *Muehlenbeckia astonii* |  | |  |
| P.w. = 0.63 | *Muehlenbeckia complexa* | P.w. = 0.63 | *Olearia virgate* |  | |  |
|  | *Phormium cookianum* |  | *Ozothamnus leptophyllus* |  |  | |
|  | *Pinus contorta* |  | *Phormium cookianum* |  |  | |
|  | *Pinus radiata* |  | *Podocarpus totara* |  |  | |
|  | *Poa colensoi* |  | *Sophora microphylla* |  |  | |
|  | *Podocarpus totara* |  | *Hebe odora* |  |  | |

**Table S2.** All plant species used in the experiment. Information shown includes plant species’ provenance, functional group, and the number of mesocosms (out of 80) that they were planted into.

| Plant name | Family | Provenance | Functional group | # mesocosms |
| --- | --- | --- | --- | --- |
| *Acacia dealbata* | Fabaceae | Exotic | Woody | 12 |
| *Acaena caesiiglauca* | Rosaceae | Native | Herbaceous | 20 |
| *Acaena inermis* | Rosaceae | Native | Herbaceous | 20 |
| *Achillea millefolium* | Asteraceae | Exotic | Herbaceous | 16 |
| *Agrostis capillaris* | Poaceae | Exotic | Herbaceous | 16 |
| *Alnus glutinosa* | Betulaceae | Exotic | Woody | 16 |
| *Anemanthele lessoniana* | Poaceae | Native | Herbaceous | 20 |
| *Anthoxanthum odoratum* | Poaceae | Exotic | Herbaceous | 16 |
| *Brachyglottis greyi* | Asteraceae | Native | Herbaceous | 20 |
| *Carex secta* | Cyperaceae | Native | Herbaceous | 28 |
| *Chionochloa conspicua* | Poaceae | Native | Herbaceous | 4 |
| *Cirsium vulgare* | Asteraceae | Exotic | Herbaceous | 16 |
| *Coprosma robusta* | Rubiaceae | Native | Woody | 8 |
| *Dactylis glomerata* | Poaceae | Exotic | Herbaceous | 12 |
| *Echium vulgare* | Boraginaceae | Exotic | Herbaceous | 16 |
| *Festuca novae-zelandiae* | Poaceae | Native | Herbaceous | 20 |
| *Hebe odora* | Plantaginaceae | Native | Woody | 12 |
| *Holcus lanatus* | Poaceae | Exotic | Herbaceous | 16 |
| *Hypericum perforatum* | Hypericaceae | Exotic | Herbaceous | 16 |
| *Leptospermum scoparium* | Myrtaceae | Native | Woody | 12 |
| *Lolium perenne* | Poaceae | Exotic | Herbaceous | 16 |
| *Lupinus arboreus* | Fabaceae | Exotic | Woody | 20 |
| *Medicago sativa* | Fabaceae | Exotic | Herbaceous | 12 |
| *Muehlenbeckia astonii* | Polygonaceae | Native | Woody | 12 |
| *Muehlenbeckia complexa* | Polygonaceae | Native | Herbaceous | 12 |
| *Olearia virgata* | Asteraceae | Native | Woody | 12 |
| *Ozothamnus leptophyllus* | Asteraceae | Native | Herbaceous | 28 |
| *Phormium cookianum* | Asphodelaceae | Native | Herbaceous | 32 |
| *Pinus contorta* | Pinaceae | Exotic | Woody | 20 |
| *Pinus radiata* | Pinaceae | Exotic | Woody | 20 |
| *Poa cita* | Poaceae | Native | Herbaceous | 20 |
| *Poa colensoi* | Poaceae | Native | Herbaceous | 16 |
| *Podocarpus totara* | Podocarpaceae | Native | Woody | 12 |
| *Rumex acetosella* | Polygonaceae | Exotic | Herbaceous | 16 |
| *Rumex obtusifolius* | Polygonaceae | Exotic | Herbaceous | 16 |
| *Sophora microphylla* | Fabaceae | Native | Woody | 12 |
| *Trifolium pratense* | Fabaceae | Exotic | Herbaceous | 16 |
| *Trifolium repens* | Fabaceae | Exotic | Herbaceous | 12 |
| *Ulex europaeus* | Fabaceae | Exotic | Woody | 20 |

**Table S3.** All herbivore species used in the mesocosm experiment. Information shown includes herbivore species’ provenance (Prov.), feeding guild, degree of specialization, number of mesocosms colonized out of 80 (# meso), and whether each species self-colonized, successfully established, and reproduced within mesocosms. Y = Yes, N = No.

| Species name | Order: Family | Prov. | Guild | Specialization | # meso. | Self-colonizer | Established | Reproduced |
| --- | --- | --- | --- | --- | --- | --- | --- | --- |
| *Costelytra giveni* | Coleoptera: Scarabaeidae | Native | Root/leaf chewer | Polyphagous | 55 | N | Y | N |
| *Naupactus godmanni* | Coleoptera: Curculionidae | Exotic | Root/leaf chewer | Polyphagous | 1 | Y | Y | N |
| *Sitona obsoletus* | Coleoptera: Curculionidae | Exotic | Root/leaf chewer | Oligophagous | 20 | N | Y | N |
| *Sitona discoideus* | Coleoptera: Curculionidae | Exotic | Leaf chewer | Oligophagous | 12 | N | Y | N |
| *Lema cyanella* | Coleoptera: Chrysomelidae | Exotic | Leaf chewer | Oligophagous | 1 | Y | Y | N |
| *Epiphyas postvittana* | Lepidoptera: Tortricidae | Exotic | Leaf chewer | Polyphagous | 68 | N | Y | Y |
| *Ctenopseustis obliquana* | Lepidoptera: Tortricidae | Native | Leaf chewer | Polyphagous | 41 | N | Y | Y |
| *Planotortrix excessana* | Lepidoptera: Tortricidae | Native | Leaf chewer | Polyphagous | 44 | N | Y | Y |
| *Dialectica scalariella* | Lepidoptera: Gracillariidae | Exotic | Leaf miner | Oligophagous | 1 | N | Y | N |
| *Agrotis ipsilon* | Lepidoptera: Noctuidae | Native | Leaf chewer | Polyphagous | 2 | Y | Y | N |
| *Pseudocoremia suavis* | Lepidoptera: Geometridae | Native | Leaf chewer | Polyphagous | 1 | Y | Y | N |
| *Anzygina zealandica* | Hemiptera: Cicadellidae | Native | Sucker | Polyphagous | 79 | N | Y | Y |
| *Philaenus spumarius* | Hemiptera: Aphrophoridae | Exotic | Sucker | Polyphagous | 74 | N | Y | N |
| *Acyrthosiphon pisum* | Hemiptera: Aphidide | Exotic | Sucker | Oligophagous | 3 | Y | Y | Y |
| *Rhopalosiphum padi* | Hemiptera: Aphidide | Exotic | Sucker | Polyphagous | 16 | N | Y | Y |
| *Myzus persicae* | Hemiptera: Aphidide | Exotic | Sucker | Polyphagous | 17 | N | Y | Y |
| *Aulacorthum solani* | Hemiptera: Aphidide | Exotic | Sucker | Polyphagous | 32 | N | Y | Y |
| *Paprides nitidus* | Orthoptera: Acrididae | Native | Leaf chewer | Polyphagous | 80 | N | Y | N |
| *Sminthurus viridis* | Collembola: Sminthuridae | Exotic | Leaf chewer | Polyphagous | 53 | Y | Y | Y |
| *Deroceras* sp. | Stylommatophora: Agriolimacidae | Exotic | Leaf chewer | Polyphagous | 59 | Y | Y | Y |
| *Teleogryllus commodus* | Orthoptera: Gryllidae | Exotic | Leaf chewer | Polyphagous | 0 | N | N | - |
| *Wiseana copularis* | Lepidoptera: Hepialidae | Native | Leaf chewer | Polyphagous | 0 | N | N | - |

**Supplementary Methods**

**Experimental design and setup**

Each community was planted in a 125 L steel pot, with a bottom layer of 22 L of gravel to aid drainage out of the open bottom, 88 L of pasteurized soil and sand (50:50 mixture), and a top layer of 12 L of soil inoculum. Communities were planted with one of 20 unique communities of eight plant species (Table S3) selected from a pool of 39 plant species that co-occur in New Zealand grassland communities (19 natives, 20 exotics, Table S1). Plant species were selected based on their occurrence at sites where inoculum soil was collected, and communities were designed to vary orthogonally in their proportion of exotic and woody species (0-100 % and 0-63 %, respectively, Table S3). Plants were grown from seed or cuttings collected from New Zealand’s South Island (see Waller *et al.* 2020 for propagation details) and seedlings were randomly positioned in a ring, equally spaced around the centre of the pot during March 2017. Consistent positioning of plant species was used for replicates within each plant community, with plant communities replicated four times, and with replicates arranged together to minimise any environmental gradients.

**Herbivore cage design**

Herbivore cages (Fig. S3) were constructed using Cropsafe Protection Mesh (0.58 mm, 15% shade factor) from Cosio Industries (Auckland, New Zealand), designed to keep out small insects like aphids and psyllids. The mesh was cut and sewn into tubes (255 cm long, 81 cm diameter) with Dabond 25/V92 UV-resistant thread from Coats Industrial (Auckland, New Zealand). The tube shape was reinforced by threading No. 8 wire (4 mm diameter) through loops sewn 75 cm from the top and bottom of the mesh. One open end of each tube was tightly drawn together and closed with cable ties, then hung from an overhead wire. The open bottom of each cage was secured around the mesocosm pot with two bungee cords that were later replaced by 10 cm wide strips of steel closed with a bolt. For access to the mesocosm community, we cut a 50 cm vertical slit in one side of the cage that was closed by tightly folding the mesh over on itself and secured with three 50 mm foldback binder clips.

**Herbivore collection, establishment and sampling**

Grass grub, *Costelytra giveni* Coca-Abia & Romero-Samper:

Collections of emerging adult grass grubs were made at dusk on 10 and 23 November 2017 from a garden near Southbridge, New Zealand (43°48' S, 172°15' E). Sex of the adult grass grubs was determined following Kelsey (1965)^1^ and Kain (1972)^2^, and three females and a single male (due to a natural 3:1 biased sex ratio) were added to the center of each mesocosm the day after each sampling occasion. Females were assumed to have mated at the time of introduction because of the frequent mating observed in the collection containers. Because grass grub adults are short-lived and larvae feed belowground, sampling could only be conducted during the final mesocosm harvest by thoroughly searching through plant roots and the mesocosm soil as it was homogenized. Grass grub larvae were found in 55 of the 80 mesocosms they were introduced to.

Fuller's rose weevil, *Naupactus godmanni* (Crotch):

Fuller’s rose weevil was a self-colonizer of one mesocosm and was only detected as larvae at harvest by thoroughly searching through plant roots and the mesocosm soil as it was homogenized.

Clover root weevil, *Sitona obsoletus* (Gmelin) and Lucerne weevil, *Sitona discoideus* Gyllenhal:

On 30-31 May 2017, clover root weevils and lucerne weevils were collected using a vacuum sampler from patches of clover (*Trifolium* sp.) and a field of lucerne (*Medicago sativa*), respectively, that were adjacent to the experimental site. Because a significant proportion of adult weevils are likely to be parasitized by a biological control agent (*Microctonus aethiopoides*, Hymenoptera: Braconidae), they were maintained for three weeks to purge parasitized individuals, before four weevils (two of each sex, determined by examining the shape of the posterior ventrite following Bright (1994)^3^) were added to the center of each mesocosm. Because direct feeding of adults was difficult to observe, their presence was determined by characteristic match-head size notches on clover leaf margins. Clover root weevil and lucerne weevil were detected in 20 and 12 mesocosms, respectively.

Thistle leaf beetle, *Lema cyanella* (L.):

Thistle leaf beetle is an exotic biological control agent that self-colonized three mesocosms, where it fed exclusively on Scotch thistle (*Cirsium vulgare*) and was found in low abundance as larvae only.

Light brown apple moth, *Epiphyas postvittana* (Walker):

Light brown apple moths were obtained from a colony maintained at Plant and Food Research in Auckland, New Zealand. Four 3rd instar caterpillars were added to the center of each mesocosm on 27 April 2017. A second introduction of three more caterpillars (of varying instar) was made on 6 June 2017 to supplement the low success of the first introduction. Due to the low success of both caterpillar introductions, two pairs of mated adult leafrollers were added during 17-23 October 2017. Moths were sent to Lincoln University as pupae and reared in containers, before pairs were moved to plastic cups, left to mate overnight, and then added to mesocosms. Surveys were conducted by systematically searching plants for caterpillars or the characteristic damage associated with leafrollers (i.e., webbing and rolling). Leaf rolls were gently examined and caterpillars of each leafroller species identified based on a combination of characters (i.e., size, head capsule color, and body color and patterning). Light brown apple moths successfully established in 68 mesocosms.

Brown-headed leafroller, *Ctenopseustis obliquana* (Walker):

Brown-headed leafrollers were also obtained from a colony maintained at Plant and Food Research in Auckland, New Zealand, and were introduced and surveyed using similar methodology to the other leafroller species. Four 3rd instar caterpillars were added to the center of each mesocosm on 28 April 2017. Due to the low survival of introduced caterpillars, two pairs of mated adult leafrollers were added during 17-22 October 2017. Brown-headed leafrollers successfully established in 41 mesocosms.

Green-headed leafroller, *Planotortrix excessana* (Walker):

Green-headed leafrollers were also obtained from a colony maintained at Plant and Food Research in Auckland, New Zealand, and were introduced and surveyed using similar methodology to the other leafroller species. Four 3rd instar caterpillars were added to the center of each mesocosm on 28 April 2017. A second introduction of two more caterpillars (of varying instar) was made on 6 June 2017 to supplement the low success of the first introduction. Due to the low success of both caterpillar introductions, two pairs of mated adult leafrollers were added during 17-18 October 2017. Green-headed leafrollers successfully established in 44 mesocosms.

*Echium* leaf miner*, Dialectica scalariella* (Zeller):

*Echium* leaf miner is an exotic biological control agent that attacks multiple species in the plant family Boraginaceae, and has been recorded feeding on 4 host plant species in New Zealand based on the Plant-SyNZ Database. This species was collected as leaf mines in various stages of development (from larvae to pupae) from *Echium vulgare* plants at Balmoral Lookout in Hurunui, New Zealand (42°52' S, 172°46' E). Leaf mines were placed in rearing cages and two adults (one of each sex) were added to each mesocosm between 7-17 June 2017. Pairs were collected from rearing cages during copulation to ensure females were mated. Plants were surveyed by carefully searching *Echium vulgare* for leaf mines. However, this species established in just a single mesocosm, where it was recorded on the expected host, *Echium vulgare*.

Greasy cutworm, *Agrotis ipsilon* (Hufnagel):

Greasy cutworm is a cosmopolitan caterpillar that self-colonized two mesocosms in low abundance.

Common forest looper, *Pseudocoremia suavis* Butler:

Common forest looper is a native moth species that self-colonized one mesocosm.

Leafhopper, *Anzygina zealandica* (Myers):

*Anzygina zealandica* is a common native leafhopper that self-colonized several mesocosms in high abundance and was subsequently identified using Knight (1976)^4^. We then added eight individuals of this species to each of the uncolonized mesocosms, collected using a sweep net from a mixture of grass species neighboring the experimental site. Plants were surveyed by systematically searching for leafhoppers, which were usually first noticed as they flew off the focal plant as it was being searched. If the host plant could not be positively identified (i.e., the leafhopper originated from a mixture of species or was on the herbivore cage) then the individual was ignored. The leafhopper successfully established in 79 mesocosms.

Meadow spittlebug, *Philaenus spumarius* (L.):

Meadow spittlebug nymphs of varying instar were collected from a range of host plants at Balmoral Lookout, Hurunui on 12 November 2017, and Birdlings Flat, Banks Peninsula (43°48' S, 172°41' E), on 21 November 2017. Five nymphs were added to the center of each mesocosm on 13 November 2017, and an additional ten nymphs were added on 21 November 2017. Plants were surveyed by systematically searching for the characteristic spittle of nymphs or by observing adults feeding on plants during surveys. Meadow spittlebugs successfully established in 74 mesocosms.

Pea aphid, *Acyrthosiphon pisum* Harris:

Pea aphids self-colonized three mesocosms where they fed on exotic legume species. Plants were surveyed by systematically examining plant tissue for aphid colonies. If less than 500 aphids were found (as was always the case for this species), they were counted as accurately as possible.

Cherry-oat aphid, *Rhopalosiphum padi* (L.):

Cherry-oat aphids self-colonized 16 mesocosms, with a small number of plants experiencing severe outbreaks. Thus, we located a source population on Yorkshire fog-grass (*Holcus lanatus*) adjacent to the experimental site, which was used to infest previously uncolonized mesocosms with five alates (winged adults) on 27 October 2017. Plants were surveyed by systematically examining plant tissue for aphid colonies. If less than 500 aphids were found, these were counted as accurately as possible. For plants with larger colonies of aphids, we estimated aphid abundance to the nearest 10 (or nearest 100 for plants with over 2000 individuals).

Green peach aphid, *Myzus persicae* (Sulzer):

Green peach aphids were collected from *Rumex obtusifolius* adjacent to the experimental site and three alates (winged adults) were added to mesocosms on 21 November 2017, successfully establishing in 17 mesocosms. Plants were surveyed by systematically examining plant tissue for aphid colonies. If less than 500 aphids were found, these were counted as accurately as possible. For plants with larger colonies of aphids, we estimated aphid abundance to the nearest 10.

Foxglove aphid, *Aulacorthum solani* (Kaltenbach):

Foxglove aphids were collected from a field of lucerne (*Medicago sativa*) adjacent to the experimental site and five alates (winged adults) were added to mesocosms on 27 November 2017, successfully establishing in 32 mesocosms. Plants were surveyed by systematically examining plant tissue for aphid colonies. If less than 500 aphids were found, these were counted as accurately as possible. For plants with larger colonies of aphids, we estimated aphid abundance to the nearest 10.

Alpine grasshopper, *Paprides nitidus* Hutton:

Alpine grasshoppers were collected from Molesworth Station in North Canterbury, New Zealand (42°27' S, 172°49' E). An adult male and female pair were first added to the center of each mesocosm during 8-30 May 2017. Replacement additions occurred after each herbivore survey when either grasshoppers were not observed or were found deceased. Surveys were conducted by systematically searching plants for grasshoppers.

Clover flea, *Sminthurus viridis* (L.):

Clover flea is an introduced springtail species from Europe with a broad host range but a preference for legumes. This species self-colonized 53 mesocosm communities, but always in low abundance. Plants were surveyed by systematically searching for clover fleas, which were usually first noticed as they hopped off the plant as it was being searched.

Garden slug, *Deroceras* sp.:

Garden slugs self-colonized 59 mesocosm communities. Surveys were conducted by systematically searching plants for slugs.

Black field cricket*, Teleogryllus commodus* (Walker):

Black field crickets were purchased from Inzect Direct (Wairarapa, New Zealand) and a single pair of male and female adults were introduced to mesocosms on 27 October 2017. However, this species failed to establish due to a late frost soon after their introduction.

Porina moth, *Wiseana copularis* (Meyrick):

Porina moth eggs were obtained from adult moths collected from pasture near Invermay, New Zealand (45°50' S, 170°22' E) in January 2017, and were stored at 4°C until the experiment was set up. We scattered 0.02 g of eggs (~250 eggs) throughout each mesocosm on 28 April 2017. However, we had no success in establishing this herbivore species.

**Supplementary References**

^1^Kelsey, J.M. Note on morphological differences between sexes of adult *Costelytra zealandica* (White). *New Zeal. J. Sci.* **8**, 173 (1965).

^2^Kain, W.M. Identification of the sexes in *Costelytra zealandica* (Coleoptera: Scarabaeidae). *New Zeal. J. Sci.* **15**, 85–87 (1972).

^3^Bright, D.E. Revision of the genus *Sitona* (Coleoptera: Curculionidae) of North America. *Ann. Entomol. Soc. Am.* **87**, 277–306 (1994).

^4^Knight, W.J. Typhlocybinae of New Zealand (Homoptera: Cicadellidae). *New Zeal. J. Zool.* **3**, 71-87 (1976).
